# MultiQC: summarize analysis results for multiple tools and samples in a single report

**DOI:** 10.1093/bioinformatics/btw354

**Published:** 2016-06-16

**Authors:** Philip Ewels, Måns Magnusson, Sverker Lundin, Max Käller

**Affiliations:** ^1^Department of Biochemistry and Biophysics, Science for Life Laboratory, Stockholm University, Stockholm 106 91, Sweden,; ^2^Department of Molecular Medicine and Surgery, Science for Life Laboratory, Center for Molecular Medicine, Karolinska Institutet, Stockholm, Sweden; ^3^Science for Life Laboratory, School of Biotechnology, Division of Gene Technology, Royal Institute of Technology, Stockholm, Sweden

## Abstract

**Motivation:** Fast and accurate quality control is essential for studies involving next-generation sequencing data. Whilst numerous tools exist to quantify QC metrics, there is no common approach to flexibly integrate these across tools and large sample sets. Assessing analysis results across an entire project can be time consuming and error prone; batch effects and outlier samples can easily be missed in the early stages of analysis.

**Results:** We present MultiQC, a tool to create a single report visualising output from multiple tools across many samples, enabling global trends and biases to be quickly identified. MultiQC can plot data from many common bioinformatics tools and is built to allow easy extension and customization.

**Availability and implementation:** MultiQC is available with an GNU GPLv3 license on GitHub, the Python Package Index and Bioconda. Documentation and example reports are available at http://multiqc.info

**Contact:** phil.ewels@scilifelab.se

## 1 Introduction

Advances in next-generation sequencing are leading to an avalanche of data. Whilst opening doors to new analysis types and experimental designs, expanding sample numbers make studies increasingly vulnerable to confounding batch effects (Leek *et al.,* 2010; Meyer and Liu 2014; Taub *et al.,* 2010). Such biases are often subtle and difficult to detect and require careful quality control measures.

Most bioinformatics programs produce logs detailing their results. Dedicated QC tools such as FastQC (http://www.bioinformatics.babraham.ac.uk/projects/fastqc), Qualimap (Okonechnikov *et al.*, 2015) and RSeQC (Wang *et al.*, 2012) are excellent at highlighting potential problems in data. However, nearly all of these logs and reports are produced on a per-sample basis, requiring the user to find and compile QC results. This process is time consuming, repetitive and complex, making it prone to errors.

**Fig. 1.**
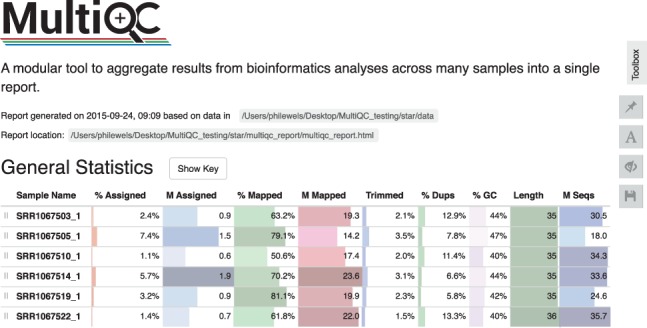
Top of a typical MultiQC report. The general statistics table can be seen with metrics from a number of different tools gathered for each sample (Color version of this figure is available at *Bioinformatics* online.)

MultiQC addresses this problem by scanning given analysis directories for log files and QC reports, creating a single summary report visualizing results across all samples. Collecting data within a single report provides a fast way to scan key statistics quickly and easily (Fig. 1). Shared plots allow accurate comparison between samples, allowing detection of subtle differences not noticeable when switching between different files. Data visualization aids batch effect detection and minimizes the risk of confounding factors affecting the results of the study. MultiQC is the first tool of its type within the field; it has the potential to greatly improve quality control and reporting for researchers involved in next-generation sequencing, removing the need for custom comparative scripts.

## 2 Materials and methods

### 2.1 Running MultiQC

MultiQC is written in Python and run on the command line; specified directories are searched recursively for any recognized files. Submodules for each supported tool are run, querying input files using configurable search settings. If any log files are found they are parsed, otherwise the module exits silently. Once all submodules have finished, the specified template is loaded and parsed using the *Jinja2* package to render the final report. Parsed data is saved as tab delimited text, YAML or JSON for downstream use. Because submodules only contribute to the report if they find logs, MultiQC is run in the same way for every analysis type. At the time of writing, MultiQC supports 22 common bioinformatics tools including aligners, processing tools and QC programs.

### 2.2 MultiQC reports

MultiQC generates a single s elf-contained HTML report which can be shared and opened in any modern web browser. Reports render plots using the JavaScript plotting library *HighCharts* (http://www.highcharts.com). Plots are resizeable and interactive, some with click and drag zooming. Samples can be renamed, hidden and highlighted using a report toolbox. Plots can be exported in a range of publication-ready formats.

Reports with hundreds of samples become too large for use with HighCharts; instead MultiQC switches to rendering plots as images at run-time using the Python plotting library MatPlotLib (Hunter, 2007). Images are embedded within the HTML, maintaining a stand-alone file with consistent file size. These static reports are also suitable for conversion to PDF using tools such as *Pandoc* (http://pandoc.org).

Each report contains an interactive walk through of features. Tutorial videos can be found at http://multiqc.info along with tutorials and documentation describing installation, usage and troubleshooting.

### 2.3 Extending MultiQC

MultiQC supports a lot of common bioinformatics tools but it is inevitable that research groups may have their own bespoke scripts or require other customization. To accommodate this, MultiQC is built in such a way that custom code can be tied into its functionality easily. Code hooks allow external plugins to access and modify the internal workings of the program. The use of Python setuptools *entry points* allows modules, templates and plugins to be kept within a separate code base, whilst still executing as part of the main MultiQC program.

Extensive documentation makes adding to MultiQC simple; four new modules have been contributed by users to date and we are aware of at least three plugins written by different research groups. Adoption by the bioinformatics community has been rapid: MultiQC has been downloaded thousands of times within the past few months and is already integrated as standard within the popular bcbio-nextgen analysis toolkit (http://bcb.io).

## 3 Typical applications

### 3.1 Single cell data and population studies

Single cell and population studies are perhaps the perfect examples of large projects where accurate quality control of numerous datasets is critical. MultiQC is able to parse data for thousands of samples within minutes, adapting report output as required. Parsed data saved by MultiQC can be used for post-processing and dataset filtering. Reports reveal overall analysis success and make it easy to identify abnormal samples.

### 3.2 Sequencing facilities

MultiQC was originally developed for use in a high throughput sequencing facility. Reports give the overview required to spot failing samples and highlighting helps to identify groups of samples behaving in an irregular manner.

Plugins allow integration with in-house systems: we have written the *MultiQC_NGI* plugin which inserts meta data from our LIMS into reports and stores summary results parsed by MultiQC in our database. This functionality is enormously powerful, facilitating large scale internal data collection that would otherwise require numerous custom scripts. Templates allow report branding and reports are self-contained, making MultiQC an ideal tool for creating delivery reports.

## 4 Conclusion

As the field of next-generation sequencing matures, there are increasing numbers of bioinformatics tools producing ever more verbose descriptions of data. Integrating these statistics across tools with large sample sets is difficult and time-consuming. MultiQC can automate the parsing of this metadata, providing powerful visualizations with a simple interface. Extension and data export allow MultiQC to function as a central collection point at the terminus of analysis pipelines. Routine use can aid quality control steps early on in data processing, reducing risk of batch effects and other downstream analysis problems.
